# Ultrasound radiomics signature for predicting central lymph node metastasis in clinically node-negative papillary thyroid microcarcinoma

**DOI:** 10.1186/s13044-024-00191-x

**Published:** 2024-02-19

**Authors:** Jie Liu, Jingchao Yu, Yanan Wei, Wei Li, Jinle Lu, Yating Chen, Meng Wang

**Affiliations:** 1 Department of Head and Neck Thyroid Surgery, Cangzhou Hospital of Integrated TCM-WM·Hebei, No.31 Huanghe West Road, 061000 Cangzhou, Hebei Province China; 2 Department of TCM Internal Medicine, Cangzhou Hospital of Integrated TCM-WM·Hebei, 061000 Cangzhou, China

**Keywords:** Papillary thyroid microcarcinoma, Radiomics, Central lymph node metastasis, Nomogram, Machine learning

## Abstract

**Background:**

Whether prophylactic central lymph node dissection is necessary for patients with clinically node-negative (cN0) papillary thyroid microcarcinoma (PTMC) remains controversial. Herein, we aimed to establish an ultrasound (US) radiomics (Rad) score for assessing the probability of central lymph node metastasis (CLNM) in such patients.

**Methods:**

480 patients (327 in the training cohort, 153 in the validation cohort) who underwent thyroid surgery for cN0 PTMC at two institutions between January 2018 and December 2020 were included. Radiomics features were extracted from the US images. Least absolute shrinkage and selection operator logistic regression were utilized to generate a Rad score. A nomogram consisting of the Rad score and clinical factors was then constructed for the training cohort. Both cohorts assessed model performance using discrimination, calibration, and clinical usefulness.

**Results:**

Based on the six most valuable radiomics features, the Rad score was calculated for each patient. A multivariate analysis revealed that a higher Rad score (*P* < 0.001), younger age (*P* = 0.006), and presence of capsule invasion (*P* = 0.030) were independently associated with CLNM. A nomogram integrating these three factors demonstrated good calibration and promising clinical utility in the training and validation cohorts. The nomogram yielded areas under the curve of 0.795 (95% confidence interval [CI], 0.745–0.846) and 0.774 (95% CI, 0.696–0.852) in the training and validation cohorts, respectively.

**Conclusions:**

The radiomics nomogram may be a clinically useful tool for the individual prediction of CLNM in patients with cN0 PTMC.

**Supplementary Information:**

The online version contains supplementary material available at 10.1186/s13044-024-00191-x.

## Introduction

Thyroid cancer remains the most common endocrine malignancy, with papillary thyroid cancer (PTC) accounting for > 90% of new cases [1–3]. Papillary thyroid microcarcinoma (PTMC) refers to PTC with a maximum size of < 10 mm, and its incidence has continually increased in recent years [4]. Although PTMC generally has excellent outcomes, patients with some risk factors (e.g., node metastases) are more likely to experience local recurrence and metastasis [5–6].

Central lymph node metastasis (CLNM) is prevalent in PTC. Even in clinically node-negative (cN0) PTMC, the rate of CLNM is high due to the low sensitivity of preoperative examinations (e.g., ultrasound [US]) [7–9]. However, whether to perform prophylactic central lymph node dissection (PCLND) in patients with cN0 PTMC remains controversial [10]. PCLND may increase postoperative morbidity, such as by causing permanent hypothyroidism [11].

Screening patients at high preoperative risk of CLNM is crucial to determining the indications for PCLND [12]. Numerous studies have analyzed the risk factors for CLNM in patients with cN0 PTMC. However, they included only limited clinicopathological and US characteristics, preventing sufficient predictive accuracy. Radiomics is a novel technology that converts imaging data into a large panel of quantitative features [13]. Here, we used US radiomics features to develop and validate a predictive model for the individualized prediction of CLNM in patients with cN0 PTMC.

## Materials and methods

### Study design

This retrospective case-control study was conducted at two institutions. Its aim was to develop and validate a model for the individual prediction of CLNM in patients with PTMC. Data were collected from 327 patients at Cangzhou Hospital of Integrated TCM-WM·Hebei for the training cohort between January 2018 and December 2020. Data were obtained from Hebei Medical University Health System (*n* = 153) during the same period for the validation cohort. The study was approved by each institution’s institutional review board. Written informed consent was obtained from all patients. This study was conducted in accordance with the Declaration of Helsinki (2013 revision) and followed the Strengthening the Reporting of Observational Studies in Epidemiology reporting guideline.

### Inclusion and exclusion criteria

The inclusion criteria were as follows: (1) age > 18 years; (2) histologically confirmed PTMC that was clinical N0 assessed by preoperative US; and (3) having undergone standard thyroidectomy and PCLND.

The exclusion criteria were as follows: (1) concurrent malignant disease of other organs; (2) recurrent or metastatic thyroid cancer; (3) history of previous surgery or radiotherapy of the neck; and (4) incomplete US or clinicopathological data (Figure S1).

### Data collection

All clinicopathological data, including age, sex, and *BRAF* status, were collected from the medical records. For the *BRAF* mutation analysis, an AmoyDx® BRAF Mutation Detection Kit (V2) (ADx-BR02; Amoy Diagnostics Co., Ltd., Xiamen, China) was utilized. The detection of the mutation was performed using a next-generation sequencing method, followed by a real-time fluorescence polymerase chain reaction–amplification refractory mutation system.

US examinations were performed using a 5–14-MHz transducer (Siemens, ACUSON Sequoia, Siemens Medical Solutions USA, Inc., Malvern, PA, USA) by radiologists with at least 8 years of experience performing thyroid US evaluations. The US characteristics of each lesion, including diameter, texture, echo, boundary rule, presence of calcification, and capsule invasion, were evaluated by an independent radiologist (W.L.).

### Feature extraction and selection

US images were retrieved from the picture archiving and communication system (Carestream, Toronto, Canada) for further feature extraction. The region of interest (ROI) of each lesion was manually segmented on the largest diameter image using ITK-SNAP software. To measure the interobserver agreement, all the manual segmentations were performed by two experienced radiologists who were blinded to patients’ characteristics. Moreover, one of the radiologists delineated the ROIs again after two weeks to measure the intraobserver agreement. The ROIs delineated by this radiologist in the second round were used for subsequent feature extraction. The radiomics feature extraction was performed using the open-source platform Pyradiomics (version 3.1.0). This platform allows the extraction of 851 radiomics features, which can be classified into shape features, first-order features, gray-level co-occurrence matrix features, gray-level size zone matrix features, gray-level run length matrix features, and gray-level dependence matrix features. The interclass correlation coefficient (ICC) was used to evaluate the inter- and intraobserver agreements of the feature extraction. Features with good consistency (ICC > 0.75) were subjected to further analysis.

Before the feature selection, the values of the extracted features were standardized with z scores. A three-step procedure was performed to select the robust radiomics features in the training cohort [14]. First, a univariable logistic regression analysis was performed to identify significant CLNM predictors with *P* < 0.05. Second, the Pearson correlation coefficient for each of the two features was calculated, and we excluded the one with a higher P value for those feature pairs with a strong correlation (Pearson *r* > 0.90). Overall, 107 features were screened out for the last selection. Third, the least absolute shrinkage and selection operator (LASSO) logistic regression model was performed to determine the optimal combination of radiomics features and calculate a radiomics (Rad) score by 10-fold cross-validations via the 1–standard error criteria.

### Statistical analysis

Categorical variables are expressed as frequencies and percentages and were compared using the chi-squared test. We calculated the hazard ratios (HRs) and 95% confidence intervals (CIs) of CLNM using the logistic regression model with uni- and multivariate analyses. Pearson correlation coefficients were calculated to evaluate correlations among the parameters.

To provide a quantitative tool to predict the individual probability of CLNM, we generated the radiomics nomogram based on the multivariate analysis of the training cohort. The discrimination of the nomogram was assessed using receiver operating characteristic curves by calculating the area under the curve (AUC). The model’s calibration was assessed using calibration curves by comparing the predicted and actual probability. A decision curve analysis was utilized to assess the clinical usefulness of the nomogram.

All statistical analyses were performed using SPSS software (version 22.0; IBM Corporation, Armonk, NY, USA) and R software version 4.1.3 (R Foundation for Statistical Computing, Vienna, Austria). Statistical significance was set at a two-tailed value of *P* < 0.05.

## Results

### Patients’ baseline characteristics

Between January 2018 and December 2020, 327 and 153 patients were included in the training and validation cohorts. For the training cohort, the mean age was 45.0 years (range, 18–74 years); there were 260 women (79.5%) and 67 men (30.5%). For the validation cohort, the mean age was 46.2 years (range, 23–72 years); there were 123 (80.4%) women and 30 men (19.6%). The demographic and ultrasound characteristics of the two cohorts are summarized in Table S1.

Table 1 shows the association between CLNM and the patients’ characteristics. CLNM was significantly associated with younger age (< 45 years: 60.9% vs. 44.7%, *P* = 0.006), larger tumors (≥ 7 mm: 59.1% vs. 36.9%, *P* < 0.001), the presence of calcification (56.4% vs. 39.2, *P* = 0.003), capsule invasion (12.7% vs. 5.1%, *P* = 0.014), and *BRAF* V600E mutation (68.2% vs. 53.0%, *P* = 0.009).

**Table 1:** Clinicopathological and ultrasound characteristics of patients according to central lymph node metastasis in the training cohort

Characteristic	CLNM group (*N* = 110)	Non-CLNM group (*N* = 217)	*P* value
Age, years			0.006
< 45	67 (60.9%)	97 (44.7%)	
≥ 45	43 (39.1%)	120 (55.3%)	
Sex			0.061
Female	81 (73.6%)	179 (82.5%)	
Male	29 (26.4%)	38 (17.5%)	
Tumor size, cm			< 0.001
< 0.7	45 (40.9%)	137 (63.1%)	
≥ 0.7	65 (59.1%)	80 (36.9%)	
Multifocality			0.097
No	67 (60.9%)	152 (70.0%)	
Yes	43 (39.1%)	65 (30.0%)	
Laterality			0.100
Unilateral	84 (76.4%)	182 (83.9%)	
Bilateral	26 (23.6%)	35 (16.1%)	
Margin			0.365
Smooth	34 (30.9%)	78 (35.9%)	
Irregular	76 (69.1%)	139 (64.1%)	
Calcification			0.003
Absent	48 (43.6%)	132 (60.8%)	
Presence	62 (56.4%)	85 (39.2%)	
Capsule invasion			0.014
Absent	96 (87.3%)	206 (94.9%)	
Present	14 (12.7%)	11 (5.1%)	
Hypoechoic			0.530
No	12 (10.9%)	19 (8.8%)	
Yes	98 (89.1%)	198 (91.2%)	
BRAF V600E mutation			0.009
Wild-type	35 (31.8%)	102 (47.0%)	
Mutant	75 (68.2%)	115 (53.0%)	

Data are expressed as N (%)

### Derivation of rad score

**Fig. 1:**
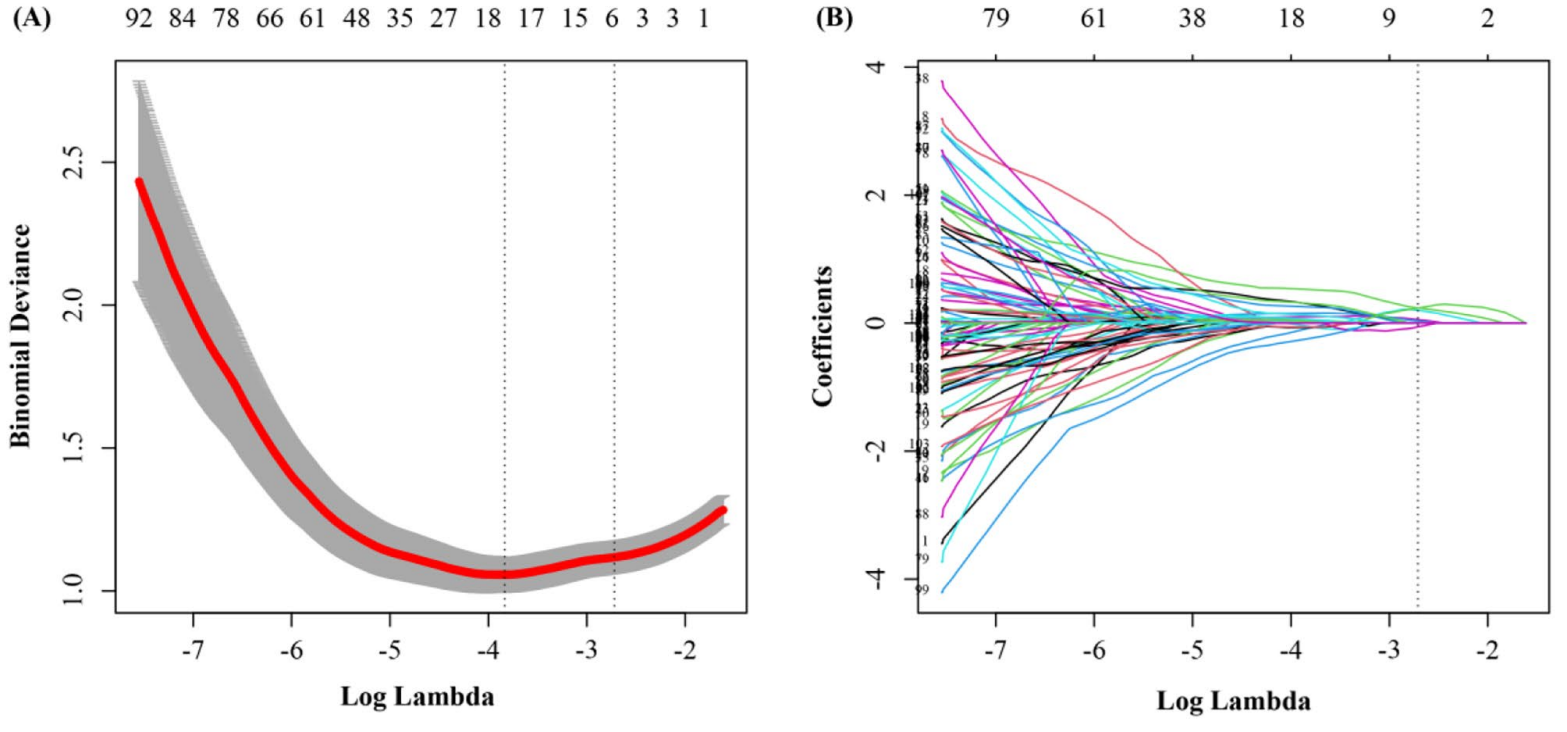
Radiomics feature selection using the least absolute shrinkage and selection operator (LASSO) logistic regression model. (**A**) Ten-fold cross-validation for tuning parameter selection in the LASSO logistic model. Solid vertical lines represent binomial deviance ± standard error. The vertical lines are drawn at the optimal values by minimum criteria and 1 - S.E. criteria. (**B**) LASSO coefficient profiles of the 107 radiomics features. A coefficient profile plot was produced against the log (λ) sequence. A vertical line was drawn at the value selected using ten-fold cross-validation, where optimal λ resulted in six nonzero coefficients

A Rad score was developed using LASSO regression analysis based on six of the 107 radiomics features in the training cohort (Fig. 1). The formula used to calculate this score is as follows:

Rad score = 0.048135878 × originalshapeMaximum2DDiameterSlice + 0.034883446 × wavelet-LLHfirstorderKurtosis + 0.016035799 × wavelet-LLHglszmGrayLevelNonUniformityNormalized + 0.007692882 × wavelet-LLLglrlmRunLengthNonUniformity + 0.051670164 × wavelet-HHLglrlmRunLengthNonUniformity - 0.009525348  × originalngtdmContrast.

**Fig. 2:**
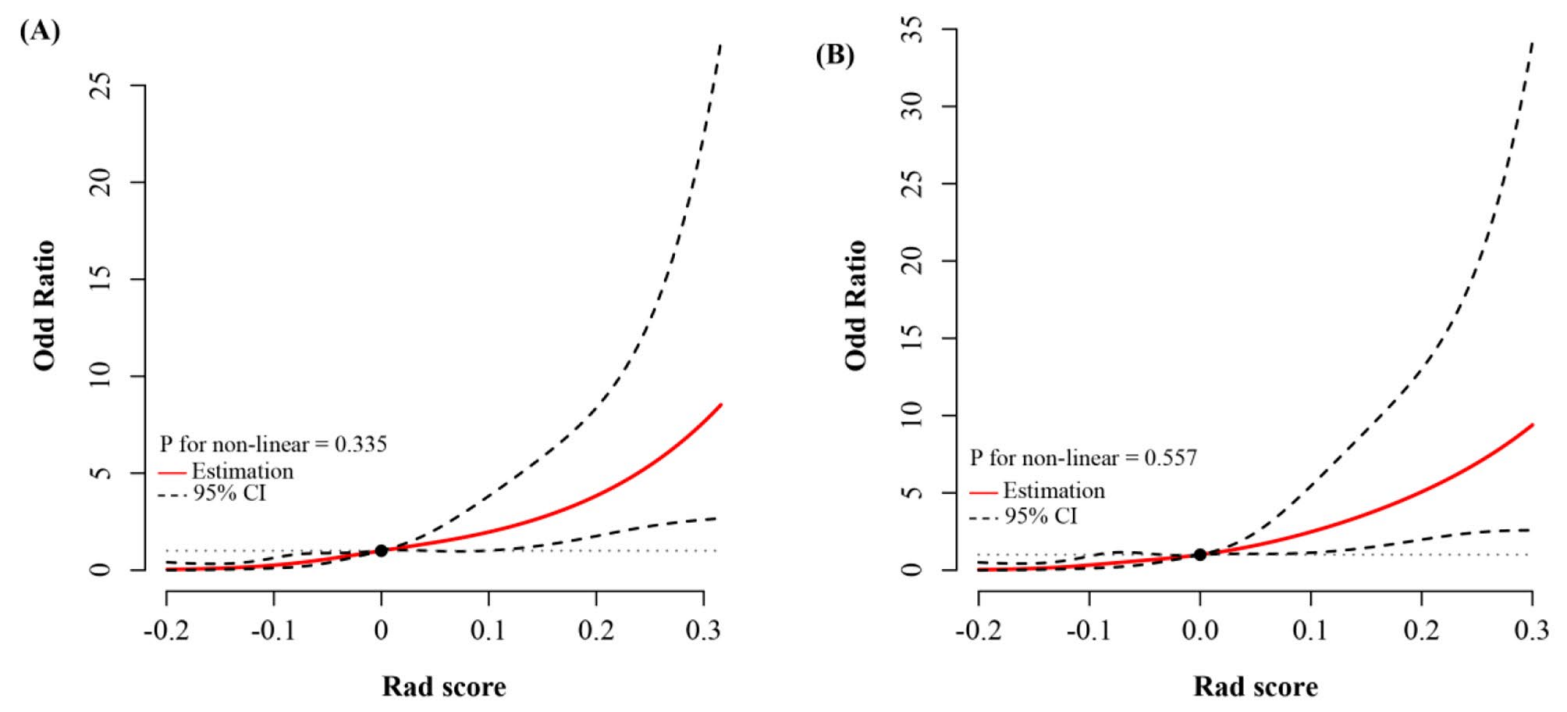
Univariate logistic analysis of central lymph node metastasis with restricted cubic splines (RCS) in the training (**A**) and validation (**B**) cohorts

In the training and validation cohorts, a significant linear relationship was observed between the Rad score and the risk of CLNM (non-linear, *P* > 0.05; Fig. 2): a higher score was associated with a higher risk of CLNM.

### Uni- and multivariate analyses

In the training cohort, the univariate analysis revealed that age, tumor size, calcification, capsule invasion, and Rad score was significantly associated with the risk of CLNM (*P* < 0.05). In the multivariate analysis, age < 45 years (HR, 0.461; 95% CI, 0.267–0.798; *P* = 0.006), presence of capsule invasion (HR, 2.885; 95% CI, 1.108–7.514; *P* = 0.030), and a higher Rad score (HR, 2.376; 95% CI, 1.843–3.063; *P* < 0.001) independently predicted the risk of CLNM (Table 2).

**Table 2:** Univariate and multivariate analyses for central lymph node metastasis in the training cohort. Abbreviations: HR, hazard ratio; CI, confidence interval

Characteristic	Univariate analysis				Multivariate analysis		
	HR	95% CI	*P* value		HR	95% CI	*P* value
Age, years							
< 45	Reference				Reference		
≥ 45	0.519	0.325–0.828	0.006		0.461	0.267–0.798	0.006
Sex							
Female	Reference						
Male	1.686	0.973–2.923	0.063				
Tumor size, cm							
< 0.7	Reference				Reference		
≥ 0.7	2.474	1.547–3.956	< 0.001		1.205	0.688–2.110	0.514
Multifocality							
No	Reference						
Yes	1.501	0.928–2.427	0.098				
Laterality							
Unilateral	Reference						
Bilateral	1.610	0.911–2.845	0.101				
Margin							
Smooth	Reference						
Irregular	1.254	0.768–2.048	0.365				
Calcification							
Absent	Reference				Reference		
Presence	2.006	1.260–3.194	0.003		1.496	0.869–2.575	0.146
Capsule invasion							
Absent	Reference				Reference		
Present	2.731	1.196–6.238	0.017		2.885	1.108–7.514	0.030
Hypoechoic							
No	Reference						
Yes	1.158	0.530–2.528	0.713				
Rad score (per 0.1 increment)	2.481	1.948–3.160	< 0.001		2.376	1.843–3.063	< 0.001

### Association between Rad score and *BRAF* status

Rad score was significantly associated with *BRAF* status in the training (Pearson *r* = 0.313, *P* < 0.001) and validation (Pearson *r* = 0.256, *P* = 0.001) cohorts. After the adjustment for age, capsule invasion, and *BRAF* status, the Rad score remained independently associated with the risk of CLNM (HR, 2.316; 95% CI, 1.826–2.938; *P* < 0.001; Table S2). Moreover, the predictive accuracy of the Rad score was not associated with *BRAF* status in either cohort (Figure S2).

### Nomogram construction

**Fig. 3:**
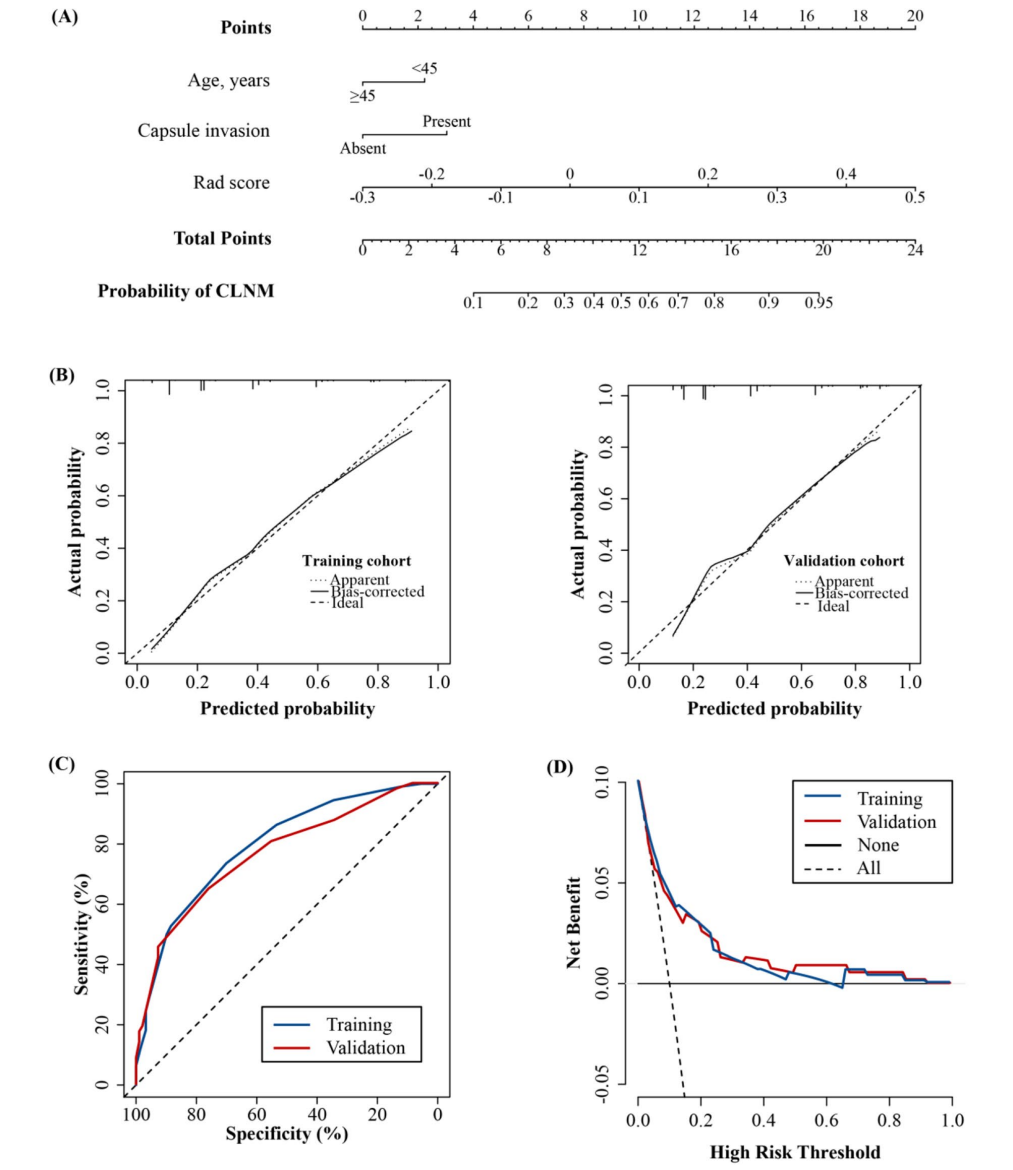
(**A**) A nomogram combining the Rad score, age, and capsule invasion for predicting probability of central lymph node metastasis (CLNM). (**B**) Plots depict the calibration of the nomogram in terms of agreement between predicted probability and actual probability in the training and validation cohorts. (**C**) Areas under the receiver operating characteristic curves for CLNM in the training and validation cohorts. (**D**) Decision curve analysis for the nomogram in the training and validation cohorts

A nomogram that combined the Rad score and other independent predictors was established to quantitatively predict the probability of CLNM (Fig. 3A).

### Model performance

The AUC values of the Rad score for predicting CLNM were 0.768 (95% CI, 0.714–0.821) and 0.745 (95% CI, 0.665–0.826), respectively, in the training and validation cohorts (Table 3). The AUC value of the nomogram reached 0.795 (95% CI, 0.745–0.846) in the training cohort and 0.774 (95% CI, 0.696–0.852) in the validation cohort (Fig. 3C; Table 3). Calibration curves showed that the nomogram had a good fit for CLNM in the training and validation cohorts (Fig. 3B). Moreover, the nomogram demonstrated promising clinical utility for most risk thresholds (Fig. 3D).

**Table 3:** Predictive performance for central lymph node metastasis in the training and validation cohorts

Model	Group	AUC	Sensitivity	Specificity	PPV	NPV	TP	FP	TN	FN
Rad score	Training cohort	0.768	0.845	0.544	0.484	0.874	93	99	118	17
	Validation cohort	0.745	0.772	0.573	0.518	0.809	44	41	55	13
Nomogram	Training cohort	0.795	0.736	0.700	0.555	0.840	81	65	152	29
	Validation cohort	0.774	0.667	0.760	0.623	0.793	38	23	73	19

**Abbreviations**: AUC, area under the curve; PPV, positive predictive value; NPV, negative predictive value; TP, true positive; FP, false positive; TN, true negative; FN, false negative

## Discussion

This is the first study to explore the value of US radiomics features for predicting CLNM in patients with cN0 PTMC. The six feature–based Rad score exhibited a significant association with the risk of CLNM. Moreover, combining this score with other clinical and US factors, the nomogram provided strong predictive power in the training and validation cohorts. These results suggest that this radiomics-based predictive model is a noninvasive, objective, and reliable tool for the preoperative prediction of CLNM.

Radiomics, an emerging algorithm that translates unseen aspects of images into a readable value, has been utilized to predict nodal metastasis in various malignancies [15–17]. Radiomics enables the noninvasive assessment of intratumor heterogeneity and facilitates a better understanding of tumor behavior [18]. For example, Yan et al. established a radiomics score based on US images, which showed good performance for predicting CLNM among patients with PTC [19]. Wang et al. developed a computed tomography radiomics signature for the preoperative prediction of CLNM in PTC [20]. In the present study, the Rad score was independently associated with the risk of CLNM, confirming the radiomics signature’s value for assessing intratumoral heterogeneity. For patients with PTMC and a higher Rad score, more aggressive treatments, such as PCNLD, should be recommended, even among those with cN0 disease.

This study incorporated Rad score and conventional clinical and US characteristics into a nomogram as in a previous study [13]. In addition to the Rad score, age and capsule invasion were independent risk factors for CLNM. In most previous studies, younger age was closely correlated with a high incidence of CLNM irrespective of the threshold [12, 21–22]. This may be explained by the negative association between age and PTMC promotion rate [23]. Capsule invasion is defined as tumor cells that invade the thyroid capsule, contributing to a high risk of CLNM [12, 21–22]. A feasible explanation for this is that the tumor could more easily metastasize to the central lymph nodes upon breaching the capsule [24].

Molecular alterations associated with *BRAF* mutations influence PTMC initiation, progression, and metastasis. Some studies reported a high incidence of CLNM in patients with PTMC and *BRAF* mutations [21, 25]. In the present study, *BRAF* status was also significantly associated with the risk of CLNM. Moreover, the predictive accuracy of the Rad score was not influenced by *BRAF* status, confirming its value.

Some limitations of this study should be discussed. First, this was a retrospective study with a small sample size, which may lead to selection bias. Second, although the Rad score was verified in an external cohort, external validation cohorts from other countries were lacking to confirm its generalizability. Third, the AUC values of the radiomics nomogram were lower than we expected, indicating that the radiomics data from only grayscale US images were insufficient. Multimodal US images, such as contrast-enhanced and elastography, are needed in further studies.

## Conclusions

In conclusion, the nomogram constructed based on US radiomics features combined with clinical and US characteristics is a reliable tool with high accuracy for predicting CLNM in patients with cN0 PTMC to enable the tailoring of individualized treatment strategies for them. A prospective international large-scale study must further validate this predictive model.

### Electronic supplementary material

Below is the link to the electronic supplementary material.

Supplementary Material 1

## Data Availability

The datasets used and/or analyzed during the current study are available from the corresponding author on reasonable request.

## References

[1] Sung H, Ferlay J, Siegel RL (2021). Global Cancer statistics 2020: GLOBOCAN estimates of incidence and Mortality Worldwide for 36 cancers in 185 countries. CA Cancer J Clin.

[2] Lim H, Devesa SS, Sosa JA, Check D, Kitahara CM (2017). Trends in thyroid Cancer incidence and mortality in the United States, 1974–2013. JAMA.

[3] Zhang MN, Liang XY, Li MT (2023). Current status and temporal trend of disease burden of thyroid cancer in China from 1990 to 2019. Asia Pac J Clin Oncol.

[4] Cabanillas ME, McFadden DG, Durante C (2016). Thyroid cancer. Lancet.

[5] Yu XM, Wan Y, Sippel RS, Chen H (2011). Should all papillary thyroid microcarcinomas be aggressively treated? An analysis of 18,445 cases. Ann Surg.

[6] Pedrazzini L, Baroli A, Marzoli L, Guglielmi R, Papini E (2013). Cancer recurrence in papillary thyroid microcarcinoma: a multivariate analysis on 231 patients with a 12-year follow-up. Minerva Endocrinol.

[7] Zhou YL, Gao EL, Zhang W (2012). Factors predictive of papillary thyroid micro-carcinoma with bilateral involvement and central lymph node metastasis: a retrospective study. World J Surg Oncol.

[8] Khokhar MT, Day KM, Sangal RB (2015). Preoperative High-Resolution Ultrasound for the Assessment of Malignant Central Compartment Lymph nodes in papillary thyroid Cancer. Thyroid.

[9] Liu Z, Wang R, Zhou J (2021). Ultrasound lymphatic imaging for the diagnosis of metastatic central lymph nodes in papillary thyroid cancer. Eur Radiol.

[10] Calò PG, Pisano G, Medas F (2014). Total thyroidectomy without prophylactic central neck dissection in clinically node-negative papillary thyroid cancer: is it an adequate treatment?. World J Surg Oncol.

[11] Ataş H, Akkurt G, Saylam B, Tez M (2021). Central neck dissection is an independent risk factor for incidental parathyroidectomy. Acta Chir Belg.

[12] Wang Z, Gui Z, Wang Z (2023). Clinical and ultrasonic risk factors for high-volume central lymph node metastasis in cN0 papillary thyroid microcarcinoma: a retrospective study and meta-analysis. Clin Endocrinol (Oxf).

[13] Tang J, Jiang S, Ma J (2022). Nomogram based on radiomics analysis of ultrasound images can improve preoperative BRAF mutation diagnosis for papillary thyroid microcarcinoma. Front Endocrinol (Lausanne).

[14] Huang JM, Zhuang LP, Wang HG (2023). Radiomics signature for prediction of long-term survival and recurrence patterns in patients with gastric cancer after radical gastrectomy: a multicenter study. Saudi J Gastroenterol.

[15] Bian Y, Zheng Z, Fang X (2023). Artificial Intelligence To Predict Lymph Node Metastasis at CT in Pancreatic Ductal Adenocarcinoma. Radiology.

[16] Li Y, Han D, Shen C, Duan X (2023). Construction of a comprehensive predictive model for axillary lymph node metastasis in breast cancer: a retrospective study. BMC Cancer.

[17] Fang Z, Pu H, Chen XL, Yuan Y, Zhang F, Li H (2023). MRI radiomics signature to predict lymph node metastasis after neoadjuvant chemoradiation therapy in locally advanced rectal cancer. Abdom Radiol (NY).

[18] Lambin P, Rios-Velazquez E, Leijenaar R (2012). Radiomics: extracting more information from medical images using advanced feature analysis. Eur J Cancer.

[19] Yan X, Mou X, Yang Y (2023). Predicting central lymph node metastasis in patients with papillary thyroid carcinoma based on ultrasound radiomic and morphological features analysis. BMC Med Imaging.

[20] Peng Y, Zhang ZT, Wang TT (2023). Prediction of Central Lymph Node Metastasis in cN0 papillary thyroid carcinoma by CT Radiomics. Acad Radiol.

[21] Zhu D, Wu X, Zhang L, Chen Z. Predictive Value of Ultrasound Imaging Characteristics and a BRAF V600E Nomogram for Central Lymph Node Metastasis Risk in papillary thyroid microcarcinoma. Altern Ther Health Med.2023;AT8236. 37632946

[22] Wang D, Hu J, Deng C, Yang Z, Zhu J, Su X (2023). Predictive nomogram for central lymph node metastasis in papillary thyroid microcarcinoma based on pathological and ultrasound features. Front Endocrinol (Lausanne).

[23] Cho JK, Kim JY, Jeong CY (2012). Clinical features and prognostic factors in papillary thyroid microcarcinoma depends on age. J Korean Surg Soc.

[24] Seifert R, Schäfers MA, Heitplatz B, Kerschke L, Riemann B, Noto B (2021). Minimal extrathyroid extension in papillary micro carcinoma of the thyroid is an independent risk factor for relapse through lymph node and distant metastases [published online ahead of print, 2021 Mar 26]. J Nucl Med.

[25] Zhao F, Wang P, Yu C (2023). A LASSO-based model to predict central lymph node metastasis in preoperative patients with cN0 papillary thyroid cancer. Front Oncol.

