# Supplementary material for: Ultrasound radiomics signature for predicting central lymph node metastasis in clinically node-negative papillary thyroid microcarcinoma
Source: Thyroid Res. 2024 Feb 19;17:4. doi: 10.1186/s13044-024-00191-x (PMC10875890; doi:10.1186/s13044-024-00191-x)
Supplement: Supplementary file 1 — Supplementary Material 1 [file 13044_2024_191_MOESM1_ESM.docx]

**Figure S1.** Diagram of study population.


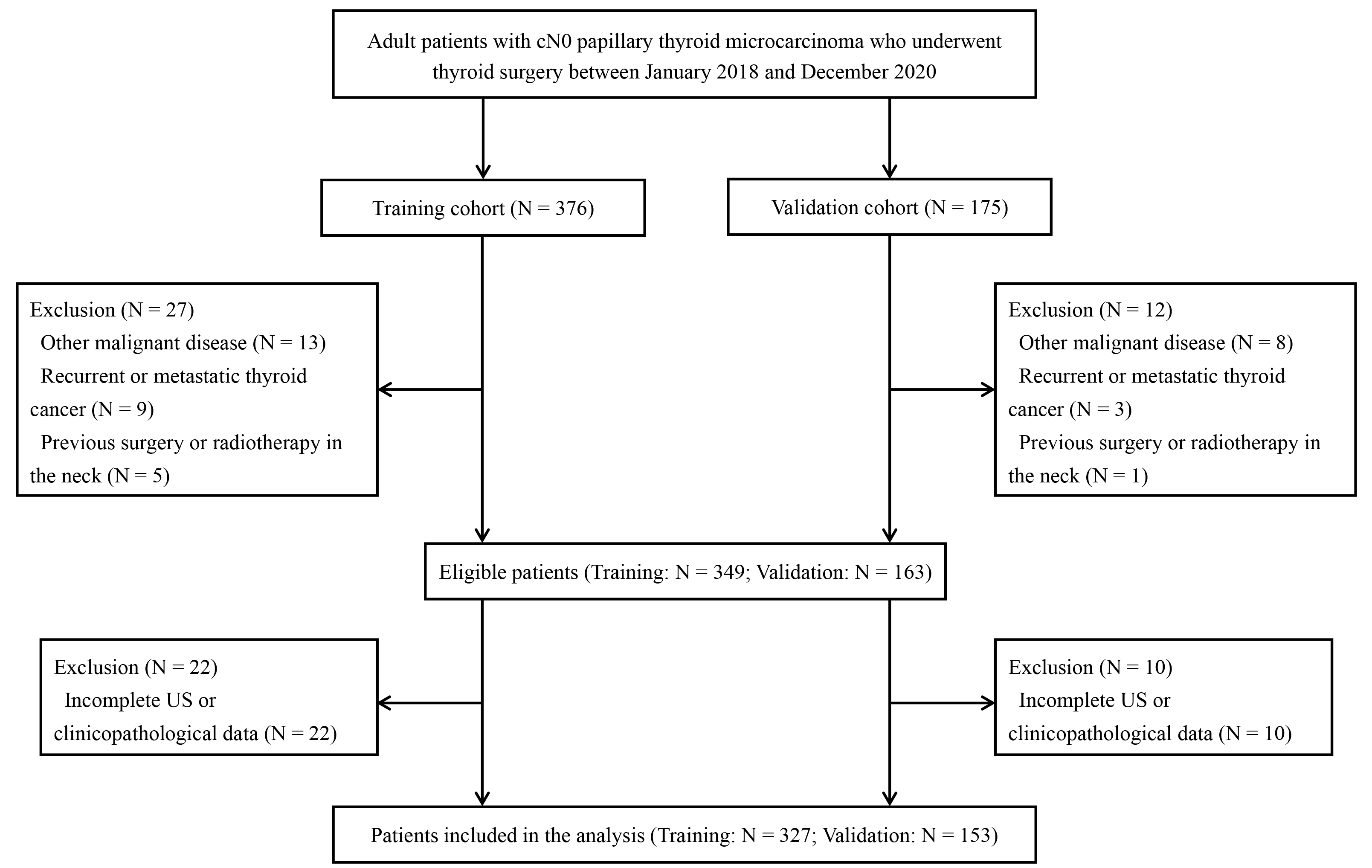


**Figure S2.** Areas under the receiver operating characteristic curves for central lymph node metastasis according to BRAF status in the training (A) and validation (B) cohorts.


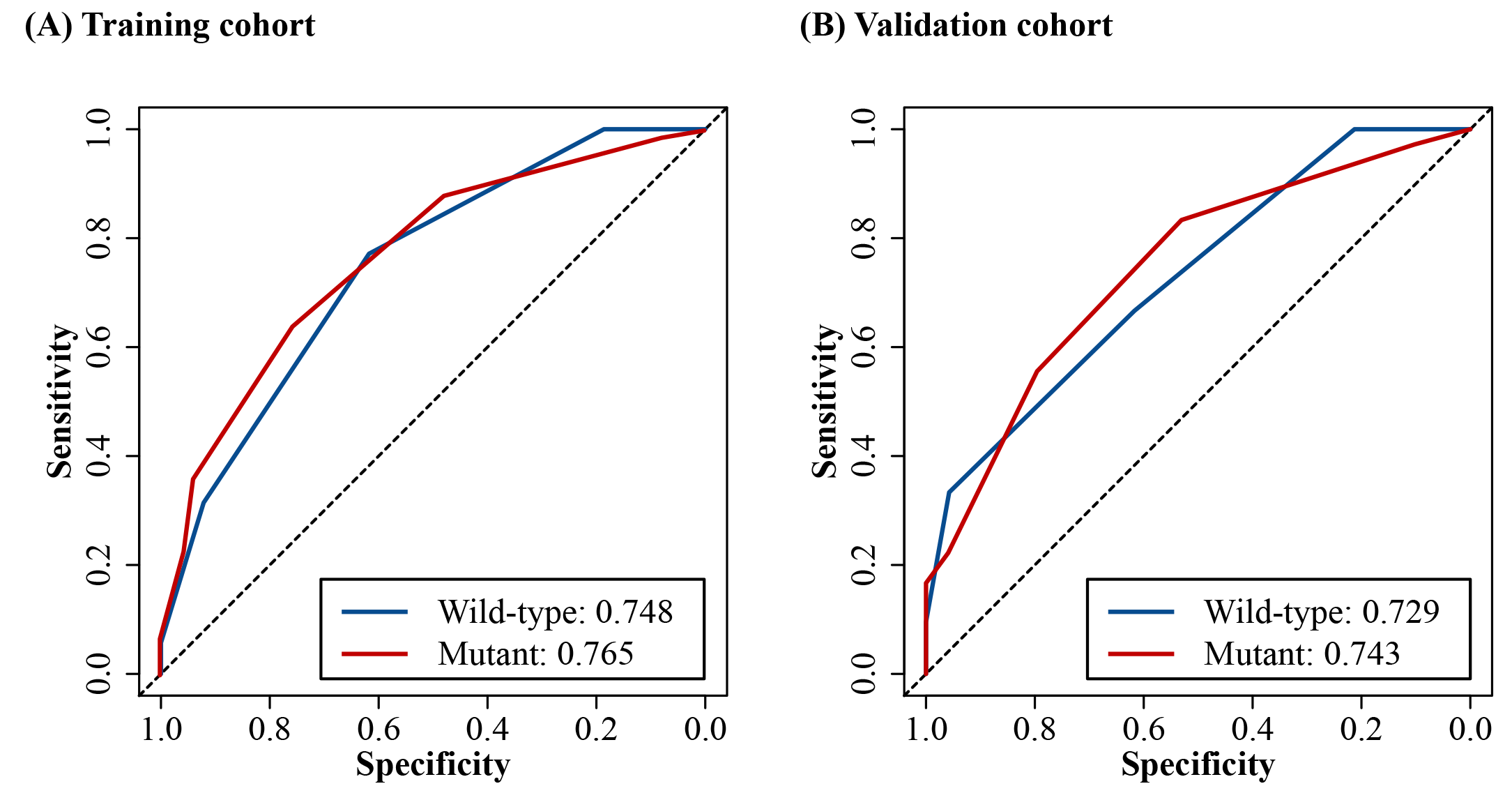


**Table S1.** Clinicopathological and ultrasound characteristics of patients in the training and validation cohorts.

| **Characteristic** | **Training cohort (N = 327)** | **Validation cohort (N = 153)** | **P value** |
| --- | --- | --- | --- |
| Age, years |  |  | 0.528 |
| < 45 | 164 (50.2%) | 72 (47.1%) |  |
| ≥ 45 | 163 (49.8%) | 81 (52.9%) |  |
| Sex |  |  | 0.823 |
| Female | 260 (79.5%) | 123 (80.4%) |  |
| Male | 67 (20.5%) | 30 (19.6%) |  |
| Tumor size, cm |  |  | 0.290 |
| < 0.7 | 182 (55.7%) | 93 (60.8%) |  |
| ≥ 0.7 | 145 (44.3%) | 60 (39.2%) |  |
| Multifocality |  |  | 0.529 |
| No | 219 (67.0%) | 98 (64.1%) |  |
| Yes | 108 (33.0%) | 55 (35.9%) |  |
| Laterality |  |  | 0.128 |
| Unilateral | 266 (81.3%) | 133 (86.9%) |  |
| Bilateral | 61 (18.7%) | 20 (13.1%) |  |
| Margin |  |  | 0.435 |
| Smooth | 112 (34.3%) | 58 (37.9%) |  |
| Irregular | 215 (65.7%) | 95 (62.1%) |  |
| Calcification |  |  | 0.612 |
| Absent | 180 (55.0%) | 88 (57.5%) |  |
| Presence | 147 (45.0%) | 65 (42.5%) |  |
| Capsule invasion |  |  | 0.748 |
| Absent | 302 (92.4%) | 140 (91.5%) |  |
| Present | 25 (7.6%) | 13 (8.5%) |  |
| Hypoechoic |  |  | 0.558 |
| No | 31 (9.5%) | 12 (7.8%) |  |
| Yes | 296 (90.5%) | 141 (92.2%) |  |
| BRAF V600E mutation |  |  | 0.599 |
| Wild-type | 137 (41.9%) | 68 (44.4%) |  |
| Mutant | 190 (58.1%) | 85 (55.6%) |  |
| CLNM |  |  | 0.438 |
| Absent | 217 (66.4%) | 96 (62.7%) |  |
| Presence | 110 (33.6%) | 57 (37.3%) |  |

**Table S2.** Multivariate analysis for central lymph node metastasis in the training cohort. Abbreviations: HR, hazard ratio; CI, confidence interval.

| **Characteristic** | **Multivariate analysis** | | |
| --- | --- | --- | --- |
|  | **HR** | **95% CI** | **P value** |
| Age (≥ 45 years) | 0.460 | 0.268-0.789 | 0.005 |
| Capsule invasion (Present) | 2.828 | 1.098-7.282 | 0.031 |
| BRAF V600E mutation (Mutant) | 1.262 | 0.729-2.183 | 0.406 |
| Rad score (per 0.1 increment) | 2.316 | 1.826-2.938 | <0.001 |
